# Serum levels of rapid turnover proteins are decreased and related to systemic inflammation in patients with ovarian cancer

**DOI:** 10.3892/ol.2013.1735

**Published:** 2013-12-06

**Authors:** TAKAFUMI WATANABE, MASAHIKO SHIBATA, HIROSHI NISHIYAMA, SHU SOEDA, SHIGENORI FURUKAWA, KENJI GONDA, SEIICHI TAKENOSHITA, KEIYA FUJIMORI

**Affiliations:** 1 Department of Obstetrics and Gynecology, Fukushima Medical University, Fukushima, Fukushima 960-1295, Japan; 2 Department of Gastrointestinal Oncology, Saitama Medical University International Medical Center, Hidaka, Saitama 350-1298, Japan; 3 Department of Tumor and Host Bioscience, Fukushima Medical University, Fukushima, Fukushima 960-1295, Japan; 4 Department of Organ Regulatory Surgery, Fukushima Medical University, Fukushima, Fukushima 960-1295, Japan

**Keywords:** ovarian cancer, malnutrition, rapid turnover protein, inflammation, cachexia, interleukin-17

## Abstract

Poor nutritional status is common in ovarian cancer. It is well known that the nutritional status of a patient with malignant disease is associated with survival, and that it can be assessed by serum levels of rapid turnover proteins (RTPs), such as retinol binding protein, prealbumin and transferrin. Systemic inflammation, usually observed in the form of elevated C-reactive protein (CRP) or neutrophil/lymphocyte ratio (NLR), occurs by various mechanisms involving numerous pro-inflammatory cytokines. These include interleukin (IL)-17 and other soluble protein mediators, such as soluble IL-2 receptor (sIL-2R) and vascular endothelial growth factor (VEGF). In this study, circulating levels of RTP were decreased in advanced stages of ovarian cancer, and significant inverse correlations were found between RTP levels and serum levels of CRP or NLR. CRP levels were also correlated with serum levels of VEGF and sIL-2R. Moreover, NLR, VEGF and sIL-2R levels, and IL-17 production, were all inversely correlated with RTP levels. These findings indicate that chronic inflammation may be associated with compromised immune function, such as an impaired T-cell response, via various inflammatory proteins, including sIL-2R, VEGF and IL-17. The key mechanisms leading to cancer cachexia, in which nutritional impairment is a major clinical issue, appear to be primarily immune reactions caused by chronic inflammation. Anti-inflammatory treatments may be effective in clinically improving various symptoms associated with these mechanisms.

## Introduction

Ovarian cancer is a heterogeneous and rapidly progressive disease of low prevalence and poor survival. It is one of the major causes of cancer-related mortality in women. In 2011, there were 4,705 deaths per 100,000 women from ovarian cancer in Japan (1). Poor nutritional status is common in ovarian cancer, and is a well-known variable that affects cancer treatment and outcomes (2). In advanced stages, it is associated with cachexia and ascites from malnutrition. The nutritional status of a patient with malignant disease is known to be associated with survival, and can be assessed by serum protein levels (2). The assessment of nutritional status is essential for a diagnosis of nutritional compromise, and measurements of serum concentrations of rapid turnover proteins (RTPs) such as retinol binding protein (RBP), prealbumin (PA) and transferrin (TF) have been reported to be more accurate for assessment than albumin (3–7).

There is increasing evidence that a systemic inflammatory response is of prognostic value in patients with various types of cancer. An elevated serum C-reactive protein (CRP) concentration is associated with a poor prognosis in colorectal, breast and ovarian cancer. Hypoalbuminemia, which is often associated with elevated CRP levels, has been reported to be a good predictor of poor prognosis in many types of cancer (8–10). Systemic inflammation occurs by various mechanisms involving numerous pro-inflammatory cytokines and other soluble protein mediators. Among them, soluble interleukin (IL)-2 receptor (sIL-2R) is part of a membrane receptor for IL-2, which can be localized on the surface of various lymphoid cells, including activated T cells, natural killer cells, monocytes, eosinophils and certain tumor cells. We have previously reported that increased production of sIL-2R is correlated with an inhibition of cell-mediated immunity, as well as with systemic inflammation and nutritional impairment, and it may be involved in immunological mechanisms that induce cancer cachexia (11,12). IL-17 is believed to stimulate various cell types to produce proinflammatory mediators that amplify intestinal inflammation, such as in inflammatory bowel diseases, or rheumatoid arthritis (13–16). The production of IL-17 has recently been reported to be associated with systemic inflammation, immune suppression and hypoalbuminemia in patients with gastrointestinal cancer (17). In ovarian cancer, we have reported that serum levels of vascular endothelial growth factor (VEGF), a glycosylated angiogenesis mediator, are elevated and correlated with malnutrition and inflammation (18).

Tumor progression has been reported to be frequently associated with systemic inflammation, and decreased serum albumin levels can be observed in this clinical scenario. In the present study, the correlation between decreased levels of RTP (including RBP, PA and TF, and CRP) and other inflammation-related proteins (such as sIL-2R, IL-17 and VEGF) was examined.

## Materials and methods

### Sample collection

Blood samples were collected from 41 patients with ovarian cancer. The patient group included four patients with stage I disease, two with stage II disease, 13 with stage III disease and 22 with stage IV disease. The enrolled patients had undergone surgery or chemotherapy at the Department of Obstetrics and Gynecology at Fukushima Medical University Hospital between May 2011 and February 2013. The patients were between 38 and 84 years old (median, 59.2 years) and newly diagnosed, with histological confirmation of the diagnosis. Blood samples were collected prior to initiation of any treatment. Peripheral blood mononuclear cells (PBMCs) were separated over Ficoll-Hypaque (Pharmacia-Biotech, Uppsala, Sweden). The isolated PBMCs were washed twice with RPMI-1640 (Wako Pure Chemical Industries Ltd., Osaka, Japan). This study was approved by the ethics committee of Fukushima Medical University (No. 1095), and written informed consent was obtained from all patients and healthy donors.

### Cytokine production assay

To measure the production of IL-17 by PBMCs, 20 ml of heparinized blood was drawn, and PBMCs were separated by a Ficoll-density gradient centrifugation procedure. A total of 10^6^ PBMCs were cultured in 1 ml of RPMI-1640 medium containing 10% heat-inactivated fetal calf serum (Gibco-BRL, St. Louis, MO, USA) and 100 μg/ml phytohemagglutinin (Sigma, Rockville, MD, USA) for 24 h under 5% CO_2_ at 37°C. After cultivation, the aliquots of the supernatant were frozen and stored at −80°C until use. The samples were then thawed and used to measure the concentrations of IL-17 by enzyme-linked immunosorbent assay (ELISA; Quantikine test kit; R&D Systems, Minneapolis, MN, USA). Test samples were used only once after thawing.

### Markers for nutritional status and chronic inflammation

In order to evaluate the nutritional condition of the patients, serum concentrations of RBP (latex agglutination immunoassay), PA (turbidimetric immunoassay) and TF (turbidimetric immunoassay) were measured. Routine hematologic investigation included a hemogram and measurement of CRP levels. Counts of neutrophils and lymphocytes, and their ratio (neutrophil/lymphocyte ratio, NLR), in the peripheral blood of patients were used as inflammation-related markers.

### Measurements of sIL-2R and VEGF

Serum concentrations of sIL-2R and VEGF were measured by ELISA (R&D Systems) according to the manufacturer’s instructions.

### Statistical analysis

Differences between the groups were evaluated using Student’s t-test. Correlations between two variables were quantified by Spearman’s rank correlation coefficient. P<0.05 was considered to indicate a statistically significant difference.

## Results

### Serum concentrations of RTPs

**Figure 1:**
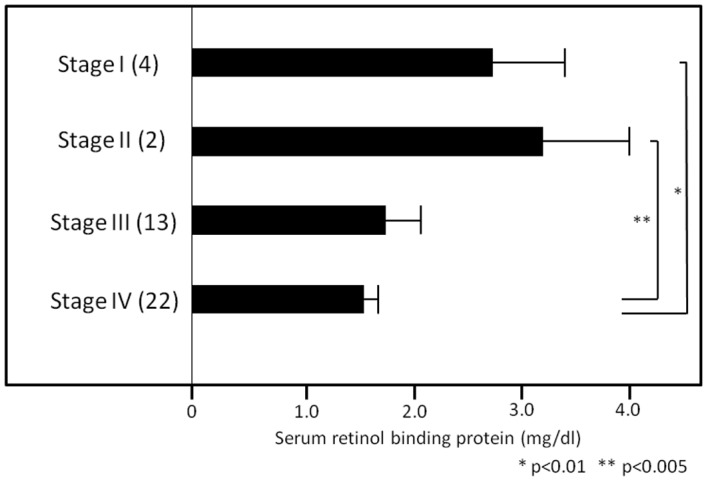
Serum levels of retinol binding protein (RBP) in patients with ovarian cancer. Serum RBP concentrations in patients with stage I, II, III and IV disease were 2.86±0.67, 3.35±0.75, 1.745±0.35 and 1.57±0.14 mg/dl, respectively. Concentrations in patients with stage IV disease were significantly lower than those in patients with stage I (P<0.01) or stage II (P<0.005) disease.

**Figure 2:**
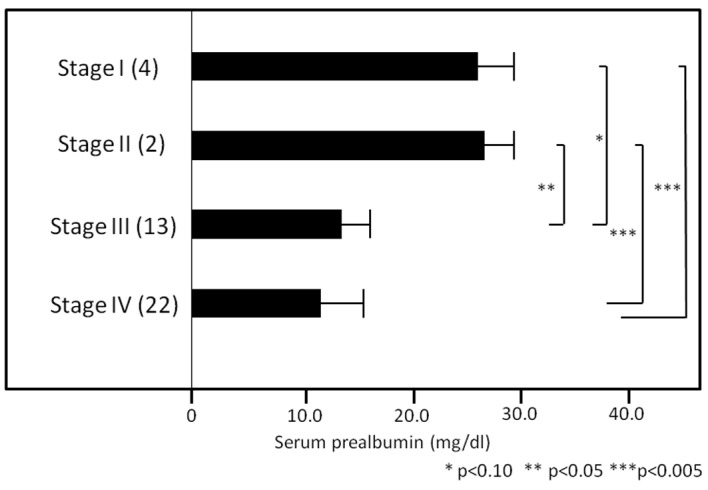
Serum levels of prealbumin (PA) in patients with ovarian cancer. Serum PA concentrations in patients with stage I, II, III and IV disease were 24.24±4.58, 25.70±2.9, 13.49±2.57 and 11.79±1.20 mg/dl, respectively. Concentrations in patients with stage IV disease were significantly lower than those in patients with stage I or II disease (both P<0.005), and concentrations in patients with stage III disease were lower than those in patients with stage II disease (P<0.05) and stage I disease (P<0.10).

**Figure 3:**
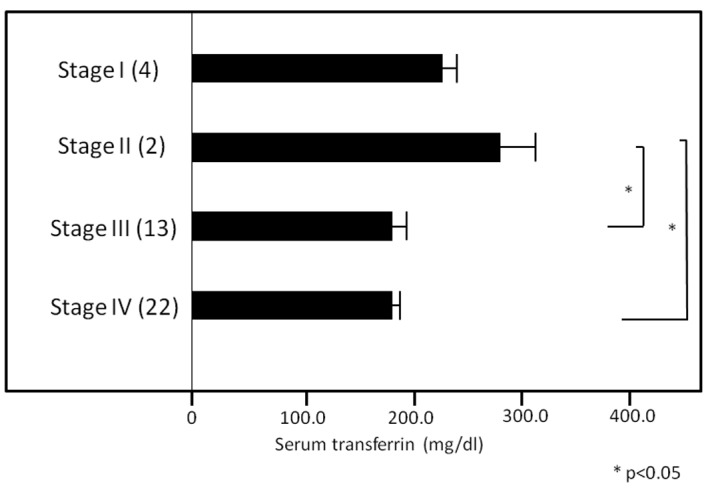
Serum levels of transferrin (TF) in patients with ovarian cancer. Serum TF concentrations in patients with stage I, II, III and IV disease were 227.2±31.3, 286.2±27.0, 179.7±20.1 and 185.8±12.5 mg/dl, respectively. Concentrations in patients with stage III or stage IV disease were lower than those in patients with stage II disease (both P<0.05).

A total of 41 samples from patients with ovarian cancer were tested. The serum RBP concentrations in patients with stage I, II, III and IV disease were 2.86±0.67, 3.35±0.75, 1.745±0.35 and 1.57±0.14 mg/dl, respectively, and were significantly lower in patients with stage IV disease than in those with stage I (P<0.01) or stage II (P<0.005) disease (Fig. 1). The serum concentrations of PA in patients with stage I, II, III and IV disease were 24.24±4.58, 25.70±2.9, 13.49±2.57 and 11.79±1.20 mg/dl, respectively. Serum PA concentrations were significantly lower in patients with stage IV disease than in those with stage I or stage II (both P<0.005, Fig. 2) disease, and patients with stage III disease had lower concentrations than those with stage II (P<0.05) and stage I (P<0.10) disease. The serum concentrations of TF in patients with stage I, II, III and IV disease were 227.2±31.3, 286.2±27.0, 179.7±20.1 and 185.8±12.5 mg/dl, respectively, and those with stage III or stage IV disease had lower concentrations than those with stage II disease (both P<0.05, Fig. 3).

### Correlations of CRP levels with RTP levels and inflammation-related proteins

Table I shows the correlations between CRP levels and serum levels of RTPs, including RBP, PA and TF, NLR as an inflammatory marker, and inflammation-related proteins, including VEGF, IL-17 and sIL-2R. Significant inverse correlations were found between CRP levels and RTP levels. NLR, VEGF levels and sIL-2R levels were significantly correlated with CRP levels.

### Correlations of serum levels of RTPs with inflammation-related factors

Table II shows the correlations between RBP, PA and TF levels, and NLR, IL-17 production, and serum VEGF and sIL-2R levels. The serum RBP concentrations showed significant inverse correlations with NLR and sIL-2R levels. PA levels were significantly and inversely correlated with NLR and sIL-2R levels, and tended to be similarly correlated with serum VEGF concentrations and IL-17 production. TF levels were significantly and inversely correlated with all factors tested in this study, including NLR, IL-17 production, and VEGF and sIL-2R concentrations.

## Discussion

Measurement of RTPs is essential in order to accurately assess nutritional status (3,4). Among these proteins, RBP, PA and TF are biologically stable and easy to measure (3,4). Cancer growth and development are associated with stimulation of the immune system, including enhanced IL-2R expression in immune cells and subsequent shedding into the circulation. A number of studies have demonstrated critical connections between clinical symptoms, survival and markers of inflammation (8–11). This study demonstrated that the circulating RTP levels were decreased in advanced stages of ovarian cancer, and that there were significant inverse correlations between RTP levels and serum CRP and NLR levels. CRP levels were also correlated with serum levels of VEGF and sIL-2R, which have been reported to be closely associated with immunosuppression and inflammation. Moreover, NLR, VEGF and sIL-2R levels, and IL-17 production, were all inversely correlated with RTP levels.

Although a causal relationship between inflammation and the innate immunity of cancer is more widely accepted today than it has been in the past, many of the precise cellular mechanisms mediating this relationship remain unclear. Increased neutrophils and decreased lymphocytes are occasionally observed in patients with advanced cancer, and NLR has been used as one of the easiest and most effective markers of chronic inflammation and related immune suppression in these patients (8,9). Malignant diseases have been found to be associated with impairment of T-cell-mediated immunity, and sIL-2R, reported to be produced primarily by lymphoid cells, appears to be crucial in this process (11). We recently noted that sIL-2R appears to be an inhibitory marker of cell-mediated immunity and nutrition (12). It was previously proposed that tumor growth and metastasis depend on angiogenesis, and blockade of angiogenesis may thus provide one strategy for inhibiting tumor growth (19). VEGF has been reported to be important in the progression of malignant neoplasms, and to induce the activity of myeloid-derived suppressor cells that appear in cancer and inflammation (18). Elevated VEGF levels are reportedly associated with advanced-stage melanoma, as well as negative immune reactions, including Th2 (type 2 helper T cell) dominance and impaired dendritic cell function. Previously reported results indicate that suppression of cell-mediated immune reactions is closely related with nutritional status, and that this appears to be involved in the development of cancer cachexia (20,21). It has recently been reported that IL-17 is important in the pathogenesis of inflammatory bowel diseases, including Crohn’s disease and ulcerative colitis. In human cancer, chronic inflammation involving IL-17 is believed to be important in the development of disease-advancement indicators, such as immune suppression or cachexia (17).

It appears that chronic inflammation may be associated with compromised immune function, such as an impaired T-cell response, via various inflammatory proteins including sIL-2R, VEGF and IL-17. It has been reported that the key mechanisms leading to cancer cachexia, in which nutritional impairment is a major clinical issue, are mostly immune reactions caused by chronic inflammation, and that treatment with a cyclooxygenase-2 inhibitor, or a specific nutrient formula, is effective (22,23). Further studies are warranted to explore this possibility and to increase the understanding of this field of medicine.

## Figures and Tables

**Table I:** Correlations of serum levels of CRP with rapid turnover proteins and inflammation-related proteins.

	Coefficient number	P-value
Retinol binding protein (mg/dl)	−0.659	2.76E-06^a^
Prealbumin (mg/dl)	−0.662	2.43E-06^a^
Transferrin (mg/dl)	−0.758	9.69E-09^a^
Neutrophil/lymphocyte ratio	0.348	0.025^a^
Serum VEGF (pg/ml)	0.522	0.00047^a^
Production of interleukin-17 (pg/ml)	−0.225	0.155
Serum soluble interleukin-2 receptor (pg/ml)	0.764	5.97E-09^a^

a Statistically significant. There were significant inverse correlations between CRP levels and levels of RTPs. Neutrophil/lymphocyte ratio, and serum VEGF and soluble interleukin-2 receptor levels were significantly correlated with CRP levels. CRP, C-reactive protein; VEGF, vascular endothelial growth factor; RTP, rapid turnover protein.

**Table II:** Correlation of rapid turnover protein with the neutrophil/lymphocyte ratio and inflammation-related proteins.

	Retinol binding protein (mg/dl)	Prealbumin (mg/dl)	Transferrin (mg/dl)
Neutrophil/lymphocyte ratio	−0.4340/0.0030^a^	−0.4490/0.0019^a^	−0.4700/0.0011^a^
Serum VEGF (pg/ml)	−0.1130/0.4570	−0.2500/0.0930	−0.3080/0.0370^a^
Production of interleukin-17 (pg/ml)	−0.0800/0.5630	−0.2730/0.0650	−0.5190/0.0002^a^
Serum soluble interleukin-2 receptor (pg/ml)	−0.3620/0.0140^a^	−0.3940/0.0060^a^	−0.4290/0.0029^a^

a Statistically significant; coefficient number/P-value. The serum concentrations of RBP showed significant inverse correlations with NLR and sIL-2R levels. PA levels were significantly and inversely correlated with NLR and sIL-2R levels, and tended to be inversely correlated with serum VEGF concentrations and IL-17 production. TF levels were significantly and inversely correlated with all of the factors tested in this study, including NLR, IL-17 production, and serum VEGF and sIL-2R concentrations. NLR, neutrophil/lymphocyte ratio; VEGF, vascular endothelial growth factor; sIL-2R, soluble interleukin-2 receptor.
